# Restricted effects of androgens on glucocorticoid signaling in the mouse prefrontal cortex and midbrain

**DOI:** 10.3389/fendo.2023.1292024

**Published:** 2024-01-18

**Authors:** Jorge Miguel Amaya, Hetty C. M. Sips, Eva M. G. Viho, Jan Kroon, Onno C. Meijer

**Affiliations:** ^1^ Department of Internal Medicine, Division of Endocrinology, Leiden University Medical Center, Leiden, Netherlands; ^2^ Einthoven Laboratory for Experimental Vascular Medicine, Leiden University Medical Center, Leiden, Netherlands

**Keywords:** androgen receptor, glucocorticoid receptor, brain, dopamine, prefrontal cortex, substantia nigra

## Abstract

Glucocorticoids are key executors of the physiological response to stress. Previous studies in mice showed that the androgen receptor (AR) influenced the transcriptional outcome of glucocorticoid treatment in white and brown adipocytes and in the liver. In the brain, we observed that chronic hypercorticism induced changes in gene expression that tended to be more pronounced in male mice. In the present study, we investigated if glucocorticoid signaling in the brain could be modulated by androgen. After chronic treatment with corticosterone, dihydrotestosterone, a combination of both, and corticosterone in combination with the AR antagonist enzalutamide, we compared the expression of glucocorticoid receptor (NR3C1, also abbreviated GR) target genes in brain regions where AR and GR are co-expressed, namely: prefrontal cortex, hypothalamus, hippocampus, ventral tegmental area and substantia nigra. We observed that androgen affected glucocorticoid signaling only in the prefrontal cortex and the substantia nigra. Dihydrotestosterone and corticosterone independently and inversely regulated expression of *Sgk1* and *Tsc22d3* in prefrontal cortex. AR antagonism with enzalutamide attenuated corticosterone-induced expression of *Fkbp5* in the prefrontal cortex and of *Fkbp5* and *Sgk1* in the substantia nigra. Additionally, in the substantia nigra, AR antagonism increased expression of *Th* and *Slc18a1*, two genes coding for key components of the dopaminergic system. Our data indicate that androgen influence over glucocorticoid stimulation in the brain is not a dominant phenomenon in the context of high corticosterone levels, but can occur in the prefrontal cortex and substantia nigra.

## 1 Introduction

Glucocorticoids (GCs) regulate many physiological processes, including energy homeostasis, peripheral circadian signaling, and adaptation to stress (1). In humans the predominant form of endogenous glucocorticoid is cortisol, whereas in mice it is corticosterone. In the brain, glucocorticoids influence diverse processes, such as neurogenesis, neuronal activity, emotional regulation and cognition (2). Glucocorticoids target both neurons and glial cells (oligodendrocytes, astrocytes and microglia), which we have previously described to undergo extensive changes in mice with chronically elevated corticosterone levels (3, 4).

Corticosterone binds to the glucocorticoid receptor (NR3C1, hereafter GR) and the mineralocorticoid receptor (MR). GR and MR belong to the superfamily of steroid nuclear receptors, which also includes the androgen receptor (AR) (5–9). Upon ligand binding, nuclear receptors homo- or heterodimerize and translocate to the cell nucleus in order to activate or repress gene transcription (10). The DNA binding domains of AR and GR are very similar, and both receptors can bind to common response elements, possibly as heterodimers (11). Modulation of glucocorticoid signaling by androgen has been demonstrated in peripheral organs, via several potential mechanisms. In liver, brown and white adipose tissues, GR-driven gene expression is potentiated when AR is activated, and such potentiation is abolished when an AR antagonist is administered (12, 13). AR is expressed in different regions of the brain, and it has been demonstrated that AR agonism in the brain influences hypothalamic pituitary adrenal (HPA) axis activity (14–16).

Previous studies addressed sex differences in the response to stressors, of which glucocorticoids are an integral part (17). However, less is known of modulation of glucocorticoid activity by sex hormones in specific brain regions or in transcriptional/translational profiles specific to particular brain cell types. Such relationships can be explored via animal models of chronic hormonal imbalances. Recently, we observed sex-dependent differences in the brain in the AdKO_2.0_ mouse (a mouse model with a deletion of the regulatory subunit of the PKA specific to the adrenal gland, which provokes adrenal enlargement and chronic hypercorticosteronemia) in comparison to wild types, including changes in brain volume, as well as in the expression of diverse mRNAs and proteins (3, 18). Notably, male mice presented larger differences than females in myelin basic protein, hippocampal aquaporin 4 and allograft inflammatory factor1 mRNA expression in cortex and hippocampus (4). We hypothesized that these findings could be explained by modulation of GC-signaling by androgens, given the sexual differences in circulating androgens and AR expression in the brain (19).

In order to explore the influence of androgen over glucocorticoid signaling in the brain, we measured the expression of a selection of genes which are responsive to GR activation by glucocorticoids. For this we selected brain areas that are known to co-express AR and GR (20). We utilized tissue from mice in which manipulations affect GR signaling in peripheral tissues (12). First, we compared mice chronically exposed to high levels of corticosterone, dihydrotestosterone, or a combination of both, to evaluate the possible potentiation effects of AR agonism on GR transcriptional activity. In addition, we investigated further AR influence on GR-driven gene expression by treating mice with corticosterone in combination with the AR antagonist enzalutamide.

## 2 Materials and methods

### 2.1 Animal studies

We previously published data from these mouse cohorts, with respect to liver transcriptome (12, 21). Male C57BL6/J mice (Jackson Laboratory) were housed in conventional cages (3-4 mice per cage) under controlled temperature and 12:12 light-dark cycle, with access to regular chow diet and water *ad libitum*. At 8 weeks of age, a slow-release pellet was subcutaneously implanted, superior to the cervical vertebrae. In experiment 1, pellets contained one of the following treatments: 100 mg cholesterol (vehicle - VEH), 20 mg corticosterone + 80 mg cholesterol (CORT), 5 mg dihydrotestosterone + 20 mg corticosterone + 75 mg cholesterol (DHT+CORT), or 5 mg dihydrotestosterone + 95 mg cholesterol (DHT). In experiment 2, pellets contained one of the following treatments: 100 mg cholesterol (VEH) or 20 mg corticosterone (Sigma-Aldrich) + 80 mg cholesterol (CORT). AR antagonist Enzalutamide (ENZ; MedChemExpress) was administered via diet supplementation (357 mg drug per kg diet; resulting in an estimated dose of 40 mg/kg/day) to a subgroup of mice implanted with CORT pellets (CORT+ENZ). After 14 days of treatment, mice were euthanized and perfused intracardially with ice cold PBS for 5 minutes. After perfusion, brains were extracted from skulls, placed on aluminum foil and snap frozen on dry ice, and stored at -80°C until further processing.

### 2.2 Brain sampling

60 µm coronal sections were obtained in a cryotome (Thermo Scientific) and micro-dissected with a coring tool (Fine Science Tools), 0.8 or 1.0 mm of diameter. Five brain regions were collected using stereotactic coordinates from Paxinos and Franklin Mouse brain atlas, namely: prefrontal cortex (PFC) (bregma 1.10 to 0.62), ventromedial hypothalamus (VMH) (bregma -1.34 to -1.94), dorsal hippocampus (HIP) (bregma -1.34 to -2.06), ventral tegmental area (VTA) and substantia nigra (SN) (bregma -2.92 to -3.80). The complete dorsal hippocampus was collected by double-punching, using the 1.0 mm needle. Samples were kept frozen until further processing. The areas were selected based on prominent coexpression of AR and GR mRNA in the mouse brain as determined by *in situ* hybridization (22).

### 2.3 Selection of GR responsive genes

We determined that we could test androgen-glucocorticoid crosstalk in the brain by measuring the expression of GR responsive genes under chronic GR activation in contexts of AR activation or inactivation by means of androgen or androgen antagonist chronic treatment. GR responsive genes were selected irrespective of their biological function, using procedure described by Viho et al. (2022) (23). Briefly, selection was made by comparing previous rodent brain and neuron studies reporting transcriptomic responses upon glucocorticoid treatment (24) to hippocampal RNA-seq data from mice previously injected with corticosterone (23) and to data from two ChIP-seq studies addressing rat brain response to corticosterone injections (25, 26), and applying the following predefined filtering criteria: GC regulation, directional consistency and genomic association to binding sites for GR, MR or both. The genes we include in the present study were validated for response to chronic corticosterone treatment as used in the present experiment.

### 2.4 RNA extraction and qPCR

Total RNA was extracted from micropunches using TriPure™ Isolation Reagent (Merck), following the manufacturer’s protocol. First strand cDNA was synthesized with M-MLV RT system (Promega), to a final concentration of 1 ng/µL cDNA. qPCR reactions were run with iQ™ SYBR® Green Supermix (Bio-Rad) in real-time PCR detection system (Bio-Rad). Expression levels were normalized to reference gene Rn18s. QuantiTect® Primer Assays were purveyed by Qiagen. All information about primers used is contained in **Supplementary Table 1**. All primer pairs were exon spanning and had amplification efficacies of > 90%.

### 2.5 Statistical analysis

Data from experiment 1 were compared by means of 2-way ANOVA, with corticosterone and DHT as factors. Data from experiment 2 were compared by 1-way ANOVA. The level of significance was set at α < 0.05. For pairwise comparison *post hoc* Tukey test was used.

## 3 Results

To facilitate comparison between the complementary experiments 1 and 2, we present data from both experiments in the same figures.

### 3.1 Regulation of AR and GR mRNA

**Figure 1:**
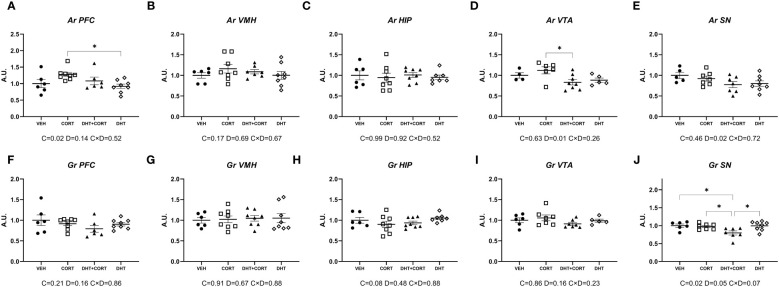
AR and Gr expression. Expression of Androgen Receptor (*Ar*, **A-E**) and Glucocorticoid Receptor (*Gr*, **F-J**) mRNA in selected brain areas, obtained from experiment 1. 2-way ANOVA main effects (p<0.05) are depicted below the x axis as: C, Corticosterone; D, Dihydrotestosterone; CxD, Interaction. *p<0.05.

As a strategy to identify effects of androgens on glucocorticoid signaling, we evaluated gene expression in brain areas that co-express AR and GR. In experiment 1, we determined the levels of mRNA expression of AR and GR, as regulation of the receptors may be a mechanism for (mutual) regulation of responsiveness. In the PFC, corticosterone increased AR mRNA expression (p=0.02, **Figure 1A**). In the ventral tegmental area and the substantia nigra, dihydrotestosterone led to a decrease in AR mRNA levels (main effect for DHT: p=0.01 and p=0.02, respectively; **Figures 1D, E**). *Gr* in the substantia nigra was significantly downregulated by corticosterone (p=0.02) and dihydrotestosterone (p=0.05) (**Figure 1J**). Posthoc analysis showed lower GR mRNA levels in DHT+CORT group compared to all the others.

Overall, AR and GR regulation was only observed in the PFC and in dopaminergic areas. In the substantia nigra corticosterone and dihydrotestosterone cooperated in the downregulation of GR.

### 3.2 Hippocampal gene expression

**Figure 2:**
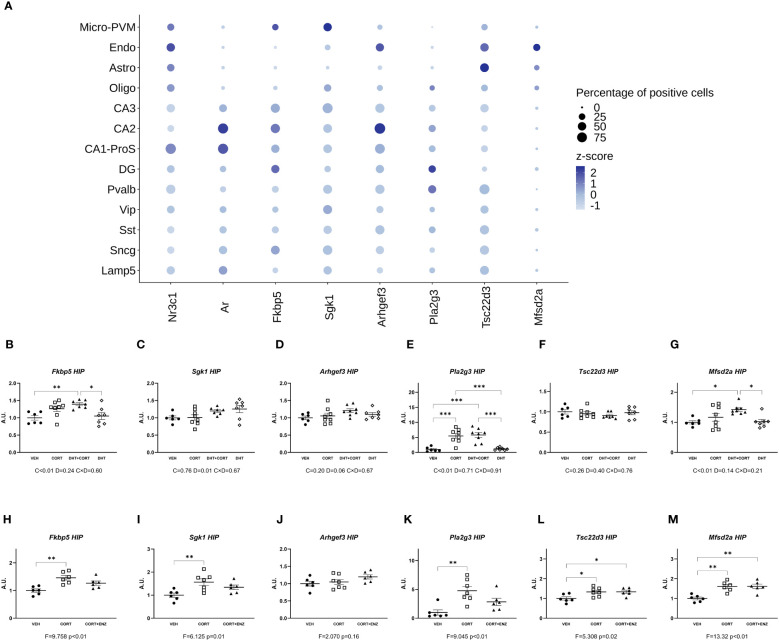
Gene expression in Hippocampus. **(A)** Normalized expression of GR, MR, AR and GR responsive genes in hippocampal cells under basal conditions. The dots represent gene centered log-normalized average expression (z-score) as color and the percentage of positive cells as dot size. Micro-PVM, microglia-perivascular macrophages; Endo, endothelial cells; Astro, astrocytes; Oligo, oligodendrocytes; CA3, CA2, CA1-ProS and DG: glutamatergic neurons from Cornu Ammonis areas 3, 2, 1 and dentate gyrus, respectively; Pvalb, Vip. Sst, Sncg and Lamp5: GABAergic neurons expressing Parvalbumin, Vasoactive Intestinal Peptide, Somatostatin, gamma-synuclein, or Lysosome-associated membrane glycoprotein 5, respectively. **(B-G)** Expression of GR responsive genes in hippocampus, obtained from experiment 1. 2-way ANOVA main effects are depicted below the x axis as: C, Corticosterone; D, Dihydrotestosterone; CxD, Interaction. **(H-M)** expression of GR responsive genes in hippocampus, obtained from experiment 2. 1-way ANOVA values of F-statistic and p are depicted below the x axis. VEH, vehicle; CORT, Corticosterone; CORT+ENZ, Corticosterone in combination with Enzalutamide. *p<0.05, **p<0.01, ***p<0.001.

The availability of a mouse hippocampal scRNA-seq dataset allowed us to determine co-expression of AR and GR in different types of cells, and this would *a priori* define potential direct effect of androgens on GC signaling. **Figure 2A** shows that AR and GR are co-expressed in neuronal cells mainly. In contrast, most of the corticosterone target genes that we selected are co-expressed with AR and GR in both neurons and non-neuronal cells. In experiment 1, corticosterone significantly increased hippocampal expression of *Fkbp5*, *Pla2g3* and *Mfsd2a* (**Figures 2B, E, G**; p<0.01 in all cases). Although no significant interaction effects with dihydrotestosterone were found, posthoc analysis showed a significant induction of *Fkbp5* and *Mfsd2a* relative to VEH only in the DHT+CORT group. *Pla2g3* was clearly induced by corticosterone, independent of dihydrotestosterone. Dihydrotestosterone, but not corticosterone, significantly increased *Sgk1* expression (p=0.01, **Figure 2C**). In experiment 2, 1-way ANOVA analyses reached statistical significance for *Fkbp5*, *Sgk1*, *Pla2g3*, *Tsc22d3* and *Mfsd2a* (p<0.05 in all cases, **Figures 2H, I, K–M**). Posthoc analyses showed that in all cases expression increased in the CORT group compared to VEH group. *Tsc22d3* and *Mfsd2a* expression increased also in the CORT +ENZ group compared to VEH group. These data indicate that gene expression in the hippocampus is responsive to GCs and DHT and that they might cooperate in the regulation of specific genes.

### 3.3 Prefrontal cortex

**Figure 3:**
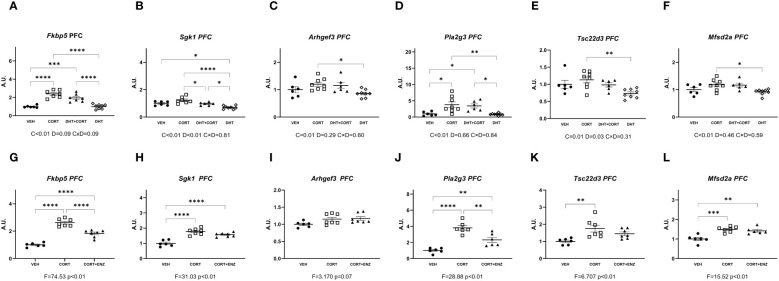
Gene expression in prefrontal cortex. **(A-F)** Expression of GR responsive genes in prefrontal cortex (PFC), obtained from experiment 1. 2-way ANOVA main effects are depicted below the x axis as: C, Corticosterone; D, Dihydrotestosterone; CxD, Interaction. **(G-L)** expression of GR responsive genes in prefrontal cortex (PFC), obtained from experiment 2. 1-way ANOVA values of F-statistic and p are depicted below the x axis. VEH, vehicle; CORT, Corticosterone; CORT+ENZ, Corticosterone in combination with Enzalutamide. *p<0.05, **p<0.01, ***p<0.001, ****p<0.0001.

In the prefrontal cortex, in experiment 1, corticosterone significantly increased mRNA expression for all genes measured (main effects p ≤ 0.01, **Figures 3A-F**). Additionally, dihydrotestosterone significantly decreased *Sgk1* and *Tsc22d3* expression (p<0.05 in both cases, **Figures 3B, E**). No interaction effects were found. Posthoc comparisons showed that for all genes the expression was significantly increased in the CORT group compared to the DHT group. *Fkbp5* and *Pla2g3* expression was also significantly higher in the CORT group compared to VEH and DHT groups. *Sgk1* expression in the DHT+CORT group was significantly lower than in the CORT group but higher than in the DHT group, and expression in the DHT group was significantly lower than in the VEH group. Overall, we found independent effects of corticosterone and dihydrotestosterone on gene expression in PFC, and these had opposite directions for those genes that were affected by both treatments.

In experiment 2, 1-way ANOVA analyses indicated statistical significance for all genes except *Arghef1* (**Figures 3G-L**). Posthoc analysis indicated that these genes were all induced by corticosterone. *Fkbp5* and *Pla2g3* expression in the CORT+ENZ group was intermediate to that in the VEH and CORT groups. *Sgk1* and *Mfsd2a* expression was increased both in CORT and CORT+ENZ groups compared to VEH group, and *Tsc22d3* expression was increased in the CORT group compared to the VEH group. These results indicate that PFC is particularly responsive to GCs, and that enzalutamide attenuated the corticosterone effects for some genes. This indicates that androgens may affect GC signaling in this brain region. However, in terms of possible mechanism, it should be noted that the AR agonist and antagonist both tended to downregulate/attenuate gene expression. Thus, there may be different mechanism in action in the two different settings that we evaluated.

### 3.4 Ventromedial hypothalamus

**Figure 4:**
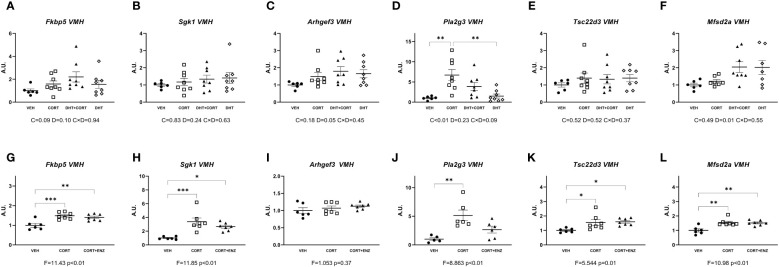
Gene expression in ventromedial hypothalamus. **(A-F)** Expression of GR responsive genes in ventromedial hypothalamus (VMH), obtained from experiment 1. 2-way ANOVA main effects are depicted below the x axis as: C, Corticosterone; D, Dihydrotestosterone; CxD, Interaction. **(G-L)** Expression of GR responsive genes in ventromedial hypothalamus (VMH), obtained from experiment 2. 1-way ANOVA values of F-statistic and p are depicted below the x axis. VEH, vehicle; CORT, Corticosterone; CORT+ENZ, Corticosterone in combination with Enzalutamide. *p<0.05, **p<0.01, ***p<0.001.

In the ventromedial hypothalamus, in experiment 1, only *Pla2g3* expression was induced by corticosterone (main effect p<0.01, **Figure 4D**). Dihydrotestosterone significantly increased *Arhgef3* and *Mfsd2a* expression (p=0.05 and p=0.01, respectively; **Figures 4C, F**). In experiment 2, 1-way ANOVA analyses indicated statistical significance for *Fkbp5*, *Sgk1*, *Pla2g3*, *Tsc22d3* and *Mfsd2a* (p ≤ 0.01 in all cases, **Figures 4G, H, J–L**). Posthoc analyses showed that *Fkbp5*, *Sgk1*, *Tsc22d3* and *Mfsd2a* expression was increased in CORT and CORT+ENZ groups compared to VEH group, and in *Pla2g3* expression was increased only in the CORT group compared to VEH group. These results suggest that in ventromedial hypothalamus most of the genes we selected are responsive to GCs. Some of these genes also are induced by dihydrotestosterone, and the effect of enzalutamide was very limited.

### 3.5 Ventral tegmental area

**Figure 5:**
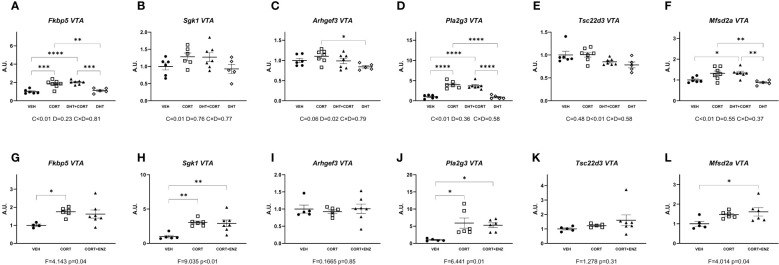
Gene expression in ventral tegmental area. **(A-F)** Expression of GR responsive genes in ventral tegmental area (VTA), obtained from experiment 1. 2-way ANOVA main effects are depicted below the x axis as: C, Corticosterone; D, Dihydrotestosterone; CxD, Interaction. **(G-L)** Expression of GR responsive genes in ventral tegmental area (VTA), obtained from experiment 2. 1-way ANOVA values of F-statistic and p are depicted below the x axis. VEH, vehicle; CORT, Corticosterone; CORT+ENZ, Corticosterone in combination with Enzalutamide. *p<0.05, **p<0.01, ***p<0.001, ****p<0.0001.

In the ventral tegmental area, in experiment 1, corticosterone significantly increased *Fkbp5*, *Sgk1*, *Pla2g3* and *Mfsd2a* expression (main effect p ≤ 0.01 in all cases; **Figures 5A, B, D, F**). Dihydrotestosterone significantly decreased *Arhgef3* and *Tsc22d3* expression (p=0.02 and p<0.01, **Figures 5C, E**). Posthoc analysis for *Fkbp5* and *Pla2g3* showed that expression was increased in the CORT and DHT+CORT groups compared to both VEH and DHT groups, while in *Arhgef3* expression was increased in the CORT group compared to the DHT group, and *Mfsd2a* expression was increased in the DHT+CORT group compared to both VEH and DHT groups, and in the CORT group compared to the DHT group. In experiment 2, 1-way ANOVA analyses reached statistical significance for *Fkbp5*, *Sgk1* and *Pla2g3* and *Mfsd2a* (p<0.05 in all cases, **Figures 5G, H, J, L**). Posthoc analyses showed increased expression of *Fkbp5* in the CORT group compared to the VEH group, increased expression of *Sgk1* and *Pla2g3* in CORT and CORT+ENZ groups compared to VEH group, and increased expression of *Mfsd2a* in CORT+ENZ group compared to VEH group.

These data show that both corticosterone and DHT are able to regulate gene expression. In experiment 1 the target genes did not overlap, and directionality of the effects was opposite. The independent effects of the hormones was confirmed in experiment 2, where enzalutamide had no effects on corticosterone-induced gene expression.

### 3.6 Substantia nigra

**Figure 6:**
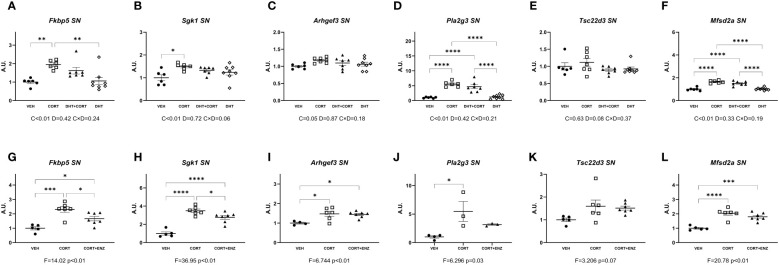
Gene expression in Substantia Nigra. **(A-F)** Expression of GR responsive genes in substantia nigra (SN), obtained from experiment 1. 2-way ANOVA main effects are depicted below the x axis as: C, Corticosterone; D, Dihydrotestosterone; CxD, Interaction. **(G-L)** Expression of GR responsive genes in substantia nigra (SN), obtained from experiment 2. 1-way ANOVA values of F-statistic and p are depicted below the x axis. VEH, vehicle; CORT, Corticosterone; CORT+ENZ, Corticosterone in combination with Enzalutamide. *p<0.05, **p<0.01, ***p<0.001, ****p<0.0001.

In the substantia nigra, in experiment 1, corticosterone significantly increased the expression of evaluated genes except for *Tsc22d3* (**Figures 6A-F**). Posthoc analyses showed that *Fkbp5* expression in the CORT group was higher than in both VEH and DHT groups. *Sgk1* expression was higher in the CORT group compared to the VEH group, and expression of *Pla2g3* and *Mfsd2a* was increased in both CORT and DHT+CORT groups compared to both VEH and DHT groups. In experiment 2, 1-way ANOVA analyses indicated statistical significance for the same genes as in experiment 1(**Figures 6G-L**). Posthoc analyses showed that *Fkbp5* and *Sgk1* expression in the CORT+ENZ group was intermediate to CORT and VEH groups. The expression of *Arhgef3* and *Mfsd2a* was increased in CORT and CORT+ENZ groups compared to the VEH group, and *Pla2g3* expression was increased in CORT group compared to VEH group.

These data show that corticosterone but not dihydrotestosterone had intrinsic effects on the selected target genes. Enzalutamide attenuated the induction of expression by corticosterone for several genes.

**Figure 7:**
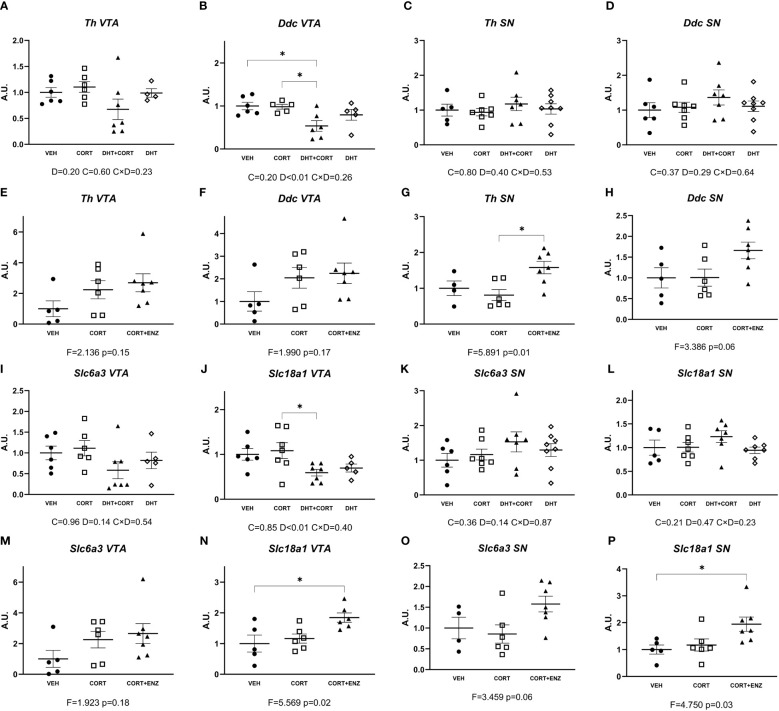
Dopamine synthesis and transport genes expression in VTA and SN. **(A–D)** Expression of dopamine synthesis genes in ventral tegmental area (VTA) and substantia nigra (SN), obtained from experiment 1. **(E–H)** Expression of dopamine synthesis genes in VTA and SN, obtained from experiment 2. **(I–L)** Expression of dopamine transport genes in VTA and SN, obtained from experiment 1. **(M–P)** Expression of dopamine transport genes in VTA and SN, obtained from experiment 2. In **(A–D)** and **(I–L)**, 2-way ANOVA main effects are depicted below the x axis as: C, Corticosterone; D, Dihydrotestosterone; CxD, Interaction. In **(E–H)** and **(M–P)**, 1-way ANOVA values of F-statistic and p are depicted below the x axis. VEH: vehicle, CORT, Corticosterone; CORT+ENZ, Corticosterone in combination with Enzalutamide. *p<0.05.

Given the availability of the tissue and the relatively direct link to physiological function, we also probed regulation of genes related to dopamine signaling in the ventral tegmental area and substantia nigra. In experiment 1, dihydrotestosterone significantly decreased expression of *Ddc* and *Slc18a1* in the ventral tegmental area (main effect p<0.01 in both cases; **Figures 7B, J**). Posthoc analysis showed that *Ddc* expression was decreased in the DHT+CORT group compared to both VEH and CORT groups, and that *Slc18a1* expression was decreased in the DHT+CORT group compared to CORT group. In experiment 2, 1-way ANOVA analyses reached statistical significance for *Slc18a1* expression in the ventral tegmental area (p=0.02, **Figure 7N**) and for the expression of *Th* and *Slc18a1* in the substantia nigra (p<0.05 in both cases, **Figures 7 G, P**). Posthoc analyses showed that *Slc18a1* expression in the ventral tegmental area was increased in the CORT+ENZ group compared to the VEH group, and that in the substantia nigra *Th* expression was increased in the CORT+ENZ group compared to the CORT group, while *Slc18a1* expression was increased in the CORT+ENZ group compared to the VEH group. Of note, the markers for dopamine signaling were the only instance in which dihydrotestosterone and enzalutamide showed opposite effects, with down-regulation by dihydrotestosterone and the occasional upregulation after enzalutamide (although the specific genes/areas were less consistent).

## 4 Discussion

In this work we aimed to test the hypothesis that androgen signaling could influence glucocorticoid signaling in the brain, which could explain our previous observation that chronic hypercorticosteronemia had more pronounced effects in male mice (3, 4) as compared to female mice. We and others had also previously observed that androgens influence GR activity in liver and adipose tissues (12, 13). Peripheral AR modulation of GR transcriptional activity was found only in regimes of chronic exposure to GCs and might be due to AR upregulation (21). Since AR-GR heterodimerization has been shown *in vitro* (11), we addressed the possibility that androgen exerts an effect over glucocorticoid signaling in brain areas where AR and GR are co-expressed (22, 27, 28).

In our experiments, the effects of androgen signaling on brain glucocorticoid-induced gene expression were modest. A priori, there are several ways by which steroid hormones may affect each other. We cannot exclude that the picture would be different in other experimental settings. For one thing, the used corticosterone treatment was earlier shown to not affect basal testosterone levels (12). However, the treatment with 20 mg pellets of corticosterone led to constant and very high levels of circulating hormone. This situation may mimic Cushing’s disease, or regimes in which high doses of synthetic glucocorticoids are used. We would expect that at these levels GRs would be likely maximally occupied, and at such saturated conditions potential modulatory effects of androgens may have been masked. And so, while our experimental design can reveal some forms of GR modulation via androgen receptors, future experiments should include more physiological hormone levels. Other limitations of our data is that we quantified bulk mRNA levels, which may have masked cell-type specific effects that may be present. Also, we did not measure protein levels, which could had helped us to infer the basal availability of AR and GR in each brain region.

Overall, we found that almost all the GR-responsive genes that we selected were upregulated by corticosterone treatment in most brain regions, while dihydrotestosterone had more limited effects. Although we observed significant effects of AR activation by dihydrotestosterone, we did not observe any statistically significant interaction effects between GC and androgens. We propose that in cases such as *Fkbp5* and *Pla2g3* in the prefrontal cortex, and *Fkbp5* and *Sgk1* in the substantia nigra, a tonic activation of AR by endogenous androgens would be enough to positively affect GR signaling, which we could unveil after blocking AR activity by means of enzalutamide. This scenario has been demonstrated previously in the study of neurobiology of sexual and aggressive behaviors (29). In our results, in the presence of enzalutamide, corticosterone treatment was still effective in upregulating *Fkbp5*, *Sgk1* and *Pla2g3* expression, yet the effect was not as pronounced when AR was blocked (**Figures 3G, J**, **6G, H**).

The fact that dihydrotestosterone and enzalutamide did not have opposite effects may also point to off-target effects of enzalutamide. These in all likelihood would not involve MR, GR or progesterone receptor (PR), given the high selectivity of enzalutamide for AR (30). Enzalutamide can induce pregnane X receptor (PXR) activity, but with an EC_50_ of more than 1µM (31). We propose that any AR independent effect of enzalutamide should therefore involve an unknown mediator. Here our conclusion is limited by the fact that we did not measure protein levels of AR and GR, as well as that we did not assess the levels of AR and GR mRNA in the experiment 2, so we cannot exclude the possibility that enzalutamide might have influenced AR and GR levels. To our knowledge this has been addressed only in prostate and bladder cancer models, with inconsistent results (32, 33). Alternative ways to further assess this would include castrating animals or the use of AR-knockout animals.

Corticosterone significantly increased the expression of *Fkbp5* in the prefrontal cortex and substantia nigra (**Figures 3A**, **6A**), and *Pla2g3* in the prefrontal cortex (**Figure 3D**) in comparison to vehicle. These increases were attenuated when corticosterone was administered in combination with enzalutamide (**Figures 3G, J**, **6G**). We had observed previously that in liver and in brown adipose tissue enzalutamide ablated the upregulation of GR target genes caused by corticosterone (12). Our data showed that in the prefrontal cortex, corticosterone treatment increased AR mRNA expression, as we have previously shown in the mouse liver (21), which suggests that AR is contributing to *Fkbp5* and *Pla2g3* upregulation upon glucocorticoid treatment. The upregulation of AR expression is the only mechanism for an effect of androgens on glucocorticoid target genes for which there is evidence in our data.

Colocalization of AR and GR in different brain areas (20, 34) would be a prerequisite for an effect of androgens on GR-mediated transcription that would depend on direct protein-protein interactions between the receptors (11). With data derived from hippocampal scRNA-seq (23, 24), we tested potential dual regulation via AR and GR for genes that would be co-expressed in particular hippocampal cell types. We found that basal co-expression of AR and GR does not *per se* predict influence of AR over GR regulation of downstream target genes. However, for hippocampal *Sgk1*, the basal expression patterns predicted gene regulation. *Sgk1* showed significant upregulation by dihydrotestosterone. This gene showed appreciable basal expression in the CA1-3 pyramidal cells, which also expressed AR (**Figure 2A**).

Outside of the hippocampus we found significant dihydrotestosterone induced upregulation of *Arhgef3* and *Mfsd2a* in the ventromedial hypothalamus (**Figures 4C, F**), and significant independent corticosterone and dihydrotestosterone effects in *Sgk1* and *Tsc22d3* in prefrontal cortex (**Figures 3B, E**). The ventromedial hypothalamus *Arhgef3* increase is probably an exclusive AR effect since corticosterone did not influence it in either experiment (**Figures 4C, I**). In the ventromedial hypothalamus increased *Mfsd2a* expression is more difficult to interpret since in experiment 1 the increase in expression is a dihydrotestosterone effect, but in experiment 2 both corticosterone and corticosterone in combination with enzalutamide increase expression (**Figures 4F, L**). This could indicate that other transcription factors interact with both GR and AR independently. In the case of the prefrontal cortex, we propose that the significant independent corticosterone and dihydrotestosterone effects in *Sgk1* and *Tsc22d3* (**Figures 3B, E**) reflect opposite transcriptional regulatory actions of AR and GR. These effects are consistent with basal expression of the receptors, and could be explained further with use of scRNA-seq data in these brain areas. Datasets recently obtained and published by Bhattacherjee et al. (35) and Hook et al. (36) could be used as a starting point for future experiments addressing possible cell-type specific functional interactions between GR and AR, be it via direct protein-protein interaction, or in more indirect ways.

Our data showed an upregulatory effect by corticosterone and a downregulatory effect by dihydrotestosterone in the prefrontal cortex on *Sgk1* and *Tsc22d3* expression. These genes are well known as shared target genes for MR, GR, and AR via established glucocorticoid response elements (GREs) in different tissues (37–39). *Sgk1* has been established as a gene upregulated by AR activation in both healthy and prostate cancer cells (40, 41). However, recently it was shown that dihydrotestosterone abolished *Sgk1* increases caused by dexamethasone stimulation in triple-negative breast cancer cells (42). This discrepancy may be related to differences in coregulator presence per cell type or at the particular chromatin locus, considering that coregulator expression varies throughout brain regions (43), and that specific configurations of coregulator expression can modify expression of nuclear receptor target genes. For example, enhancing the expression of SRC1a abolishes a dexamethasone-induced increase in *Crh* expression in the amygdala, but has no effect in *Fkbp5* (44). Given the functional diversity of the different brain regions and areas we can speculate that *Sgk1* bidirectional regulation by GR and AR is due to molecular make-up of the constituent cells of the prefrontal cortex and hippocampus.

In a previous study on steroid receptor colocalization in the mouse brain, the co-expression of GR, AR and the nuclear receptor coregulator *Pak6* in the midbrain dopaminergic regions stood out (20). Here we extended our ‘pure GR target gene’ approach with markers for dopaminergic signaling: two factors pertaining to the dopamine synthesis pathway and two dopamine transporters, in both the ventral tegmental area and the substantia nigra. Our data indicate that midbrain dopaminergic systems are regulated by androgens. We found that in ventral tegmental area, dihydrotestosterone lowered the expression of *Ddc* and *Slc18a1*. In the substantia nigra we observed that enzalutamide caused significant increases in the expression of *Th* and *Slc18a1*. Our results suggest that AR activation attenuates dopamine synthesis and presynaptic loading in the ventral tegmental area, and that its inactivation potentiates synthesis and vesicular loading. This is in accordance to previous results in which it has been observed that androgens decrease tyrosine hydroxylase (TH) expression in ventral tegmental area and substantia nigra (45) in a mechanism related to apoptosis (46, 47), as well as expression of dopamine transporter (DAT) (the translational product of *Slc6a3*) in prefrontal cortex (48). In contrast, in distinct experimental setups androgens might also exert upregulatory activities on dopaminergic system. Activated AR can increase *Th* promoter activity in cell lines (49). In several studies in adolescent male rats androgens where shown to stimulate TH and S*lc6a3* mRNA or protein expression (50–53). The discrepancies between these observations and the results obtained by us and others could be influenced by either the age of the animals or possibly by a species difference between mice and rats.

In conclusion, in our data, interplay between the glucocorticoid and androgen signaling axes in the male mouse brain was restricted to the prefrontal cortex, the substantia nigra and perhaps the hippocampus. An upregulation of AR expression by glucocorticoids is the mechanism for which there is evidence in our data, analogous to earlier observations in the liver of male mice (21). GR-driven transcription is largely resilient to AR influence, at least at the relatively high dose of corticosterone that we used. In the case of prefrontal cortex, AR activation has a downregulating effect on GR transcription of *Sgk1* and *Tsc22d3* only. Also, in both prefrontal cortex and substantia nigra, an intact AR signal appears to contribute to GR transcription of *Fkbp5* and *Sgk1*. Lastly, blockade of AR increased the expression of the dopamine-related genes *Th* and *Slc18a1*. Future works will help to clarify the influence of AR in brain cell processes and signaling routes that can explain sexual differences in health and disease.

## Data availability statement

The datasets presented in this study can be found in online repositories. The names of the repository/repositories and accession number(s) can be found below: https://osf.io/j5wtd/.

## Ethics statement

The animal study was approved by the ethical committee of Leiden University Medical Center. The study was conducted in accordance with the local legislation and institutional requirements.

## Author contributions

JA: Conceptualization, Data curation, Formal analysis, Funding acquisition, Investigation, Methodology, Project administration, Resources, Validation, Writing – original draft, Writing – review & editing. HS: Investigation, Project administration, Resources, Writing – review & editing. EV: Data curation, Writing – review & editing. JK: Conceptualization, Funding acquisition, Writing – review & editing. OM: Conceptualization, Funding acquisition, Project administration, Supervision, Writing – review & editing.
